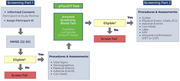# ALTITUDE‐AD: Cost Savings Using a pTau217 Screening Assay in an Ongoing Phase 2 Study of Sabirnetug in Early Alzheimer's Disease

**DOI:** 10.1002/alz70859_100249

**Published:** 2025-12-25

**Authors:** Todd Feaster, Prajakta Dinesh Mangeshkar, Siew Tin Gan, Drew Haigh, Karen Sundell, Maddelyn Hyland, Gopalan Sethuraman, Vladimir Skljarevski, June Kaplow, Robert A Dean, Jasna Jerecic, Eric Siemers

**Affiliations:** ^1^ Acumen Pharmaceuticals, Inc, Newton, MA USA; ^2^ Acumen Pharmaceuticals, Newton, MA USA

## Abstract

**Background:**

Alzheimer’s disease (AD) clinical trials exclude many potential participants who do not meet study criteria for amyloid pathology. In the phase 1 INTERCEPT‐AD study of sabirnetug (ACU193), 60% of amyloid positron emission tomography (PET) scans at screening showed negative results based on visual reads.

Because pTau217 plasma concentrations are highly predictive for AD pathology, in the ALTITUDE‐AD study (NCT06335173) we measured pTau217 at screening to identify individuals likely to show amyloid burden sufficient for study inclusion when subsequently assessed by amyloid PET or cerebrospinal fluid (CSF) Ab42/40 ratio. Individuals screened out by pTau217 would avoid lumbar punctures (LP) to collect CSF and radiation exposure from PET scans. In addition, the sponsor would avoid the associated costs.

**Methods:**

ALTITUDE‐AD is an 80‐week, global, randomized, double‐blind, placebo‐controlled phase 2 study of sabirnetug in individuals with early AD and evidence of amyloid pathology. At North American sites, screening occurred in two parts. First, blood samples were tested using the Fujirebio Lumipulse plasma pTau217 assay. Individuals with pTau217 concentrations ≥0.15 pg/mL proceeded to part two for confirmatory amyloid pathology testing. The pTau217 cut‐point was chosen for enrichment purposes and was not intended as a diagnostic tool. Total cost for the 2‐part screening was calculated for US and Canadian sites and compared with projected costs if the pTau217 cut‐point had not been used.

**Result:**

Among participants screened to date, 48% had plasma pTau217 ≥0.15 pg/mL and were eligible for confirmatory amyloid pathology testing. Overall, 81% of participants with pTau217 ≥0.15 pg/mL met amyloid burden eligibility requirements based on amyloid PET or CSF Aβ42/40 testing. Total cost of the 2‐part screening at US and Canadian sites was approximately US $15 million, whereas if the pTau217 assay had not been used, the total screening cost would have been approximately US $25 million.

**Conclusion:**

Participants tested by amyloid PET or CSF Aβ42/40 were enriched for meeting amyloid‐based inclusion criteria and the screening cost was reduced by approximately US $10 million (∼40%). Additionally, the pTau217 screening strategy helped reduce the unnecessary burden of PET scans and LP procedures for potential participants.